# Transcriptomic Profiling of Collagenous Colitis Identifies Hallmarks of Nondestructive Inflammatory Bowel Disease

**DOI:** 10.1016/j.jcmgh.2021.04.011

**Published:** 2021-04-27

**Authors:** Celia Escudero-Hernández, Atle van Beelen Granlund, Torunn Bruland, Arne Kristian Sandvik, Stefan Koch, Ann Elisabet Østvik, Andreas Münch

**Affiliations:** 1 Department of Biomedical and Clinical Sciences, Linköping University, Linköping, Sweden; 2 Department of Clinical and Molecular Medicine, Faculty of Medicine and Health Sciences, Norwegian University of Science and Technology, Trondheim, Norway; 3 Centre of Molecular Inflammation Research, Department of Clinical and Molecular Medicine, Faculty of Medicine and Health Sciences, Norwegian University of Science and Technology, Trondheim, Norway; 4 Clinic of Medicine, St Olav’s University Hospital, Trondheim, Norway; 5 Department of Gastroenterology and Hepatology, St Olav’s University Hospital, Trondheim, Norway; 6 Wallenberg Centre for Molecular Medicine, Linköping University, Linköping, Sweden; 7 Department of Gastroenterology and Hepatology, Linköping University, Linköping, Sweden; 8 Department of Health, Medicine, and Caring Sciences, Linköping University, Linköping, Sweden

**Keywords:** Epithelial Cells, Microscopic Colitis, RNA Sequencing, Ulcerative Colitis, auCC, active/untreated collagenous colitis, aRCC, active/refractory (nonresponding) collagenous colitis, CC, collagenous colitis, DEG, differentially expressed gene, DN, double negative, FDR, false discovery rate, GSEA, gene set enrichment analysis, GSVA, gene set variation analysis, IBD, inflammatory bowel disease, IEC, intestinal epithelial cell, IFN, interferon, IHC, immunohistochemistry, itCC, inactive/treated (responding) collagenous colitis, MMP, matrix metalloproteinase, PBS, phosphate-buffered saline, RNA-seq, RNA-sequencing, RT-qPCR, reverse-transcription quantitative polymerase chain reaction, TIMP, tissue inhibitor of metalloproteinase, UC, ulcerative colitis

## Abstract

**Background and Aims:**

The pathophysiology of the inflammatory bowel disease collagenous colitis (CC) is poorly described. Our aim was to use RNA sequencing of mucosal samples from patients with active CC, CC in remission, refractory CC, ulcerative colitis (UC), and control subjects to gain insight into CC pathophysiology, identify genetic signatures linked to CC, and uncover potentially druggable disease pathways.

**Methods:**

We performed whole transcriptome sequencing of CC samples from patients before and during treatment with the corticosteroid drug budesonide, CC steroid-refractory patients, UC patients, and healthy control subjects (n = 9–13). Bulk mucosa and laser-captured microdissected intestinal epithelial cell (IEC) gene expression were analyzed by gene set enrichment and gene set variation analyses to identify significant pathways and cells, respectively, altered in CC. Leading genes and cells were validated using reverse-transcription quantitative polymerase chain reaction or immunohistochemistry.

**Results:**

We identified an activation of the adaptive immune response to bacteria and viruses in active CC that could be mediated by dendritic cells. Moreover, IECs display hyperproliferation and increased antigen presentation in active CC. Further analysis revealed that genes related to the immune response (*DUOX2*, *PLA2G2A*, *CXCL9*), DNA transcription (*CTR9*), protein processing (*JOSD1, URI1*), and ion transport (*SLC9A3*) remained dysregulated even after budesonide-induced remission. Budesonide-refractory CC patients fail to restore normal gene expression, and displayed a transcriptomic profile close to UC.

**Conclusions:**

Our study confirmed the implication of innate and adaptive immune responses in CC, governed by IECs and dendritic cells, respectively, and identified ongoing epithelial damage. Refractory CC could share pathomechanisms with UC.

## Graphical abstract

**Figure:**
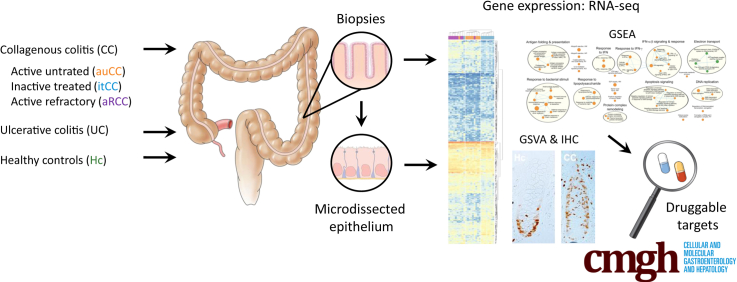

**Summary**

Collagenous colitis is a nondestructive inflammatory bowel disease that involves the innate and adaptive immune responses (ie, intestinal epithelial cell dysfunction and dendritic cell activation). The only effective treatment—the corticosteroid drug budesonide—does not fully restore gene expression.

Collagenous colitis (CC) is a debilitating inflammatory bowel disease (IBD) that causes chronic, nonbloody watery diarrhea, leading to a poor quality of life.^1^ The macroscopic appearance of the colon is usually normal, and noninvasive biomarkers do not discern between CC and other gut disorders.^1^ Thus, CC diagnosis relies on histopathological features, including a thickened collagenous band (>10 μm) and increased lymphoplasmacytic infiltrate into the lamina propria.^1^ The only effective, established treatment is the corticosteroid drug budesonide.^2^ However, clinical trials report disease relapse in 23%–39% of CC patients during maintenance treatment, and up to 80% after treatment is discontinued.^3^, ^4^, ^5^ In addition, patients can become treatment-refractory.^3^^,^^6^ Therefore, understanding CC pathogenesis is an unmet clinical need, and comprehending the mechanisms of action of budesonide would open the door for new therapeutic opportunities for patients that do not respond to treatment.

Several human leukocyte antigen (*HLA*) genetic variants have been associated with CC, which indicates activation of the adaptive immune system through antigen presentation events.^7^ As in the major IBD forms Crohn’s disease and ulcerative colitis (UC), luminal antigens could lead to the activation of an aberrant immune response.^8^ However, no association has been found between CC and pattern recognition receptor genes to date. Interestingly, nuclear factor κB, a key regulator of inflammatory immune responses involved in cytokine production, is activated in CC, specifically in intestinal epithelial cells (IECs).^9^ Therefore, these cells might play a role in CC pathogenesis.

Additional pathomechanisms compatible with an intact mucosa include changes in IEC electrophysiology and homeostasis. Transepithelial ion exchange is impaired due to low expression or loss of function of several ion channels in the colonic epithelium, leading to defects in sodium reabsorption.^10^^,^^11^ The decreased osmotic pressure impairs the paracellular reabsorption of water, which is exacerbated by the loss of the water channel aquaporin 8 in the IEC apical membrane; thus, resulting in watery diarrhea.^12^ Although ion and water channel expression are almost restored during clinical remission, the extent of epithelial dysfunction, immune responses, and dysbiosis contributing to CC pathophysiology, response to treatment, and relapse are still unclear.

It has been suggested that CC shares features with other IBDs, especially UC^1^^,^^7^^,^^13^; however, whether the similar clinical presentation of these disorders is caused by common molecular mechanisms is unclear. To address these questions, we investigated the whole transcriptome of colonic mucosa and microdissected IECs from CC patient samples. Our patient cohort, which includes budesonide-treated responding and nonresponding CC patients, has enabled us to propose targets for the development of new treatments for CC patients.

## Results

### Central Immune Response–Related Genes Are Dysregulated in CC

**Figure 1:**
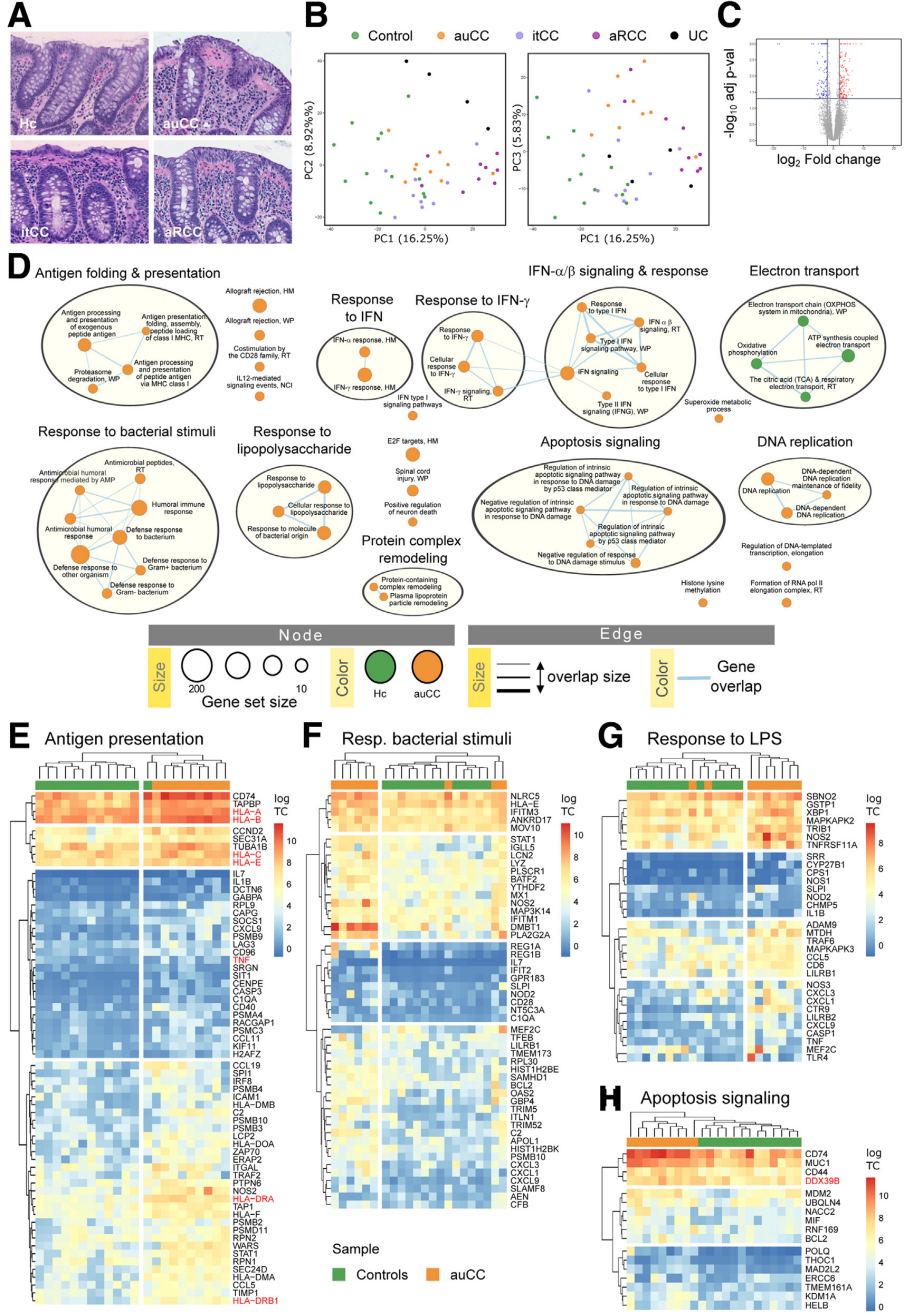
**CC mucosa gene expression indicates activation of central immune response signaling pathways.** (*A*) Representative histology of hematoxylin and eosin–stained paraffin-embedded sections of the human colonic mucosa in a healthy control (Hc) subject, an auCC patient, an itCC patient, and an aRCC patient. (*B*) Principal component analysis plot of the RNA-seq expression profiles of the different CC subgroups, Hc, and UC samples. (*C*) Volcano plot showing differentially upregulated (red) and downregulated (blue) genes in auCC compared with control subjects. (*D*) Enrichment map of gene expression in which orange nodes represent CC and green nodes represent Hc phenotype pathways created with FDR *Q* value <0.05, and combined coefficient >0.375 with combined constant = 0.5. (*E–H*) Heatmaps showing normalized log_2_-transformed fold changes (using the regularized log function in R) of RNA-seq transcript counts from leading genes contributing to the enriched gene pathways in CC colonic mucosa related to (*E*) antigen folding and presentation, (*F*) response to bacterial stimuli, (*G*) response to lipopolysaccharide (LPS), and (*H*) apoptosis signaling. n = 9–13 samples per group. Hc subjects are shown in green, auCC samples are shown in orange. Genes associated with CC by immunochip are highlighted in red. Heatmap rows and columns are split according to hierarchical clustering. Unless stated otherwise, gene pathways were retrieved from Gene Ontology Biological Process database. HM, Hallmark database; NCI, NCI-Nature curated data; RT, Reactome database; WP, WikiPathways database.

**Figure 2:**
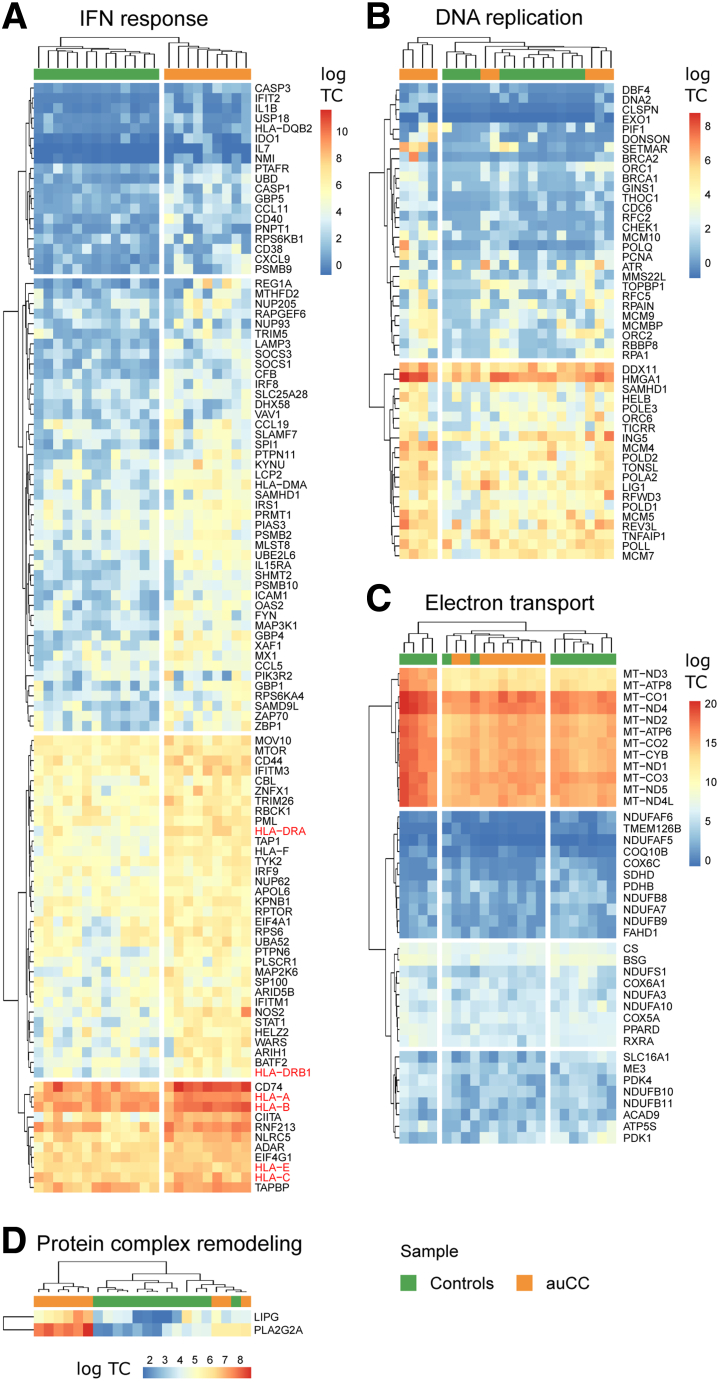
**CC mucosa displays an imbalance in IFN response, DNA replication, and metabolic processes.** (*A–D*) Heatmaps showing normalized log_2_-transformed fold changes (using the regularized log function in R) of RNA-seq transcript counts (log TC) from leading genes contributing to the enrichment of gene pathways in CC colonic mucosa displayed in Figure 1*C* related to (*A*) IFN response, (*B*) DNA replication, (*C*) electron transport, and (*D*) protein complex remodeling. Healthy control (Hc) subjects are shown in green, auCC samples are shown in orange. Genes associated with CC by immunochip^5^ are highlighted in red. n = 9–13 samples per group.

The exact pathophysiology of the chronic diarrheal disorder CC is incompletely understood. We therefore investigated the transcriptome of CC, which features an intact mucosa despite increased lymphoplasmacytic infiltration in the lamina propria (Figure 1*A*; Table 1). Principal component analyses of bulk biopsy RNA-sequencing (RNA-seq) data separated samples groups into different clusters of gene expression according to the clinical classification (Figure 1*B*). Active/untreated CC (auCC) mucosa displayed 354 differentially expressed genes (DEGs) compared with healthy control subjects (Figure 1*C*). Gene set enrichment analysis (GSEA) indicated that auCC-associated genes were related to antigen folding and presentation (*HLA*, *CD74*, *TAPBP*), response to lipopolysaccharide and bacteria (*DMBT1*, *NLRC5*, *NOS2*), apoptosis (*CD74*/*MIF*), and DNA replication events (*DDX11*, *HMGA1*) (Figure 1*D*–*H* and 2; Supplementary Table 1). A substantial number of pathways also contained genes of the response to interferons (IFNs) (Figure 1*D* and 2; Supplementary Table 1). Of note, human leukocyte antigen (*HLA*)*-I* and *-II* genes that were previously associated with CC^7^^,^^14^ have an increased expression in active CC (Figure 1, Figure 2). Collectively, our results corroborate the genetic association with *HLA*, and indicate immune response activation and bacterial recognition in CC pathogenesis.

**Table 1:** Clinical and Demographic Characteristics of the CC Patient “Exploratory” Cohort and Controls Included in RNA-Seq, Microdissected Intestinal Epithelial Cell RNA-Seq, and Immunohistochemistry Analyses

Variable	Hc	auCC	itCC^a^	aRCC	UC^b^
Total number of subjects	13^c^	9	9	9	4
On budesonide treatment	No	No	Yes	No	No
Steroid responders	—	Yes	Yes	No	—
Female, %	53.85	77.78	77.78	100	75
Average age, y	51 (17–71)	59 (27–86)	59 (27–86)	60 (25–79)	22 (19–30)
Average stools/day	—	7.56 (6–10)	1.22 (1–2)	9.89 (4–20)	—
Average watery stools/day	—	7.56 (6–10)	0 (N/A)^d^	9.89 (4–20)	—
Average collagenous band, μm	—	35.00 (16–52)	28.33 (5–72)^d^	35.56 (10–72)	—
Average stool frequency, Mayo score	—	—	—	—	2.25 (1–3)
Average endoscopy, Mayo score	—	—	—	—	2 (1–3)

NOTE. Values are n or mean (range), unless otherwise indicated.

auCC, active/untreated collagenous colitis; aRCC, active/steroid-refractory collagenous colitis; CC, collagenous colitis; Hc, healthy control subjects; itCC, inactive/treated collagenous colitis; N/A, not applicable; RNA-seq, RNA sequencing; UC, ulcerative colitis.

a Matched samples from itCC patients were collected before and during treatment with budesonide. Note that samples before treatment (active disease) were included in the group of auCC samples, whereas samples during treatment were included as itCC samples. One patient was not included for RNA-seq analysis of microdissected intestinal epithelial cells due to unavailability of paraffin-embedded biopsy sample.

b UC disease extension included 1 patient with proctitis, 2 with affection of the descending colon, and 1 with pancolitis. Patients were assessed following the Mayo score. This group was only included for bulk biopsy RNA-seq analysis.

c Nine of these patients were included for intestinal epithelial cell microdissection and subsequent RNA-seq analysis.

d Note that the average stool frequencies and collagenous band thickness before treatment of itCC patients are nearly the same as the auCC patient group.

**Figure 3:**
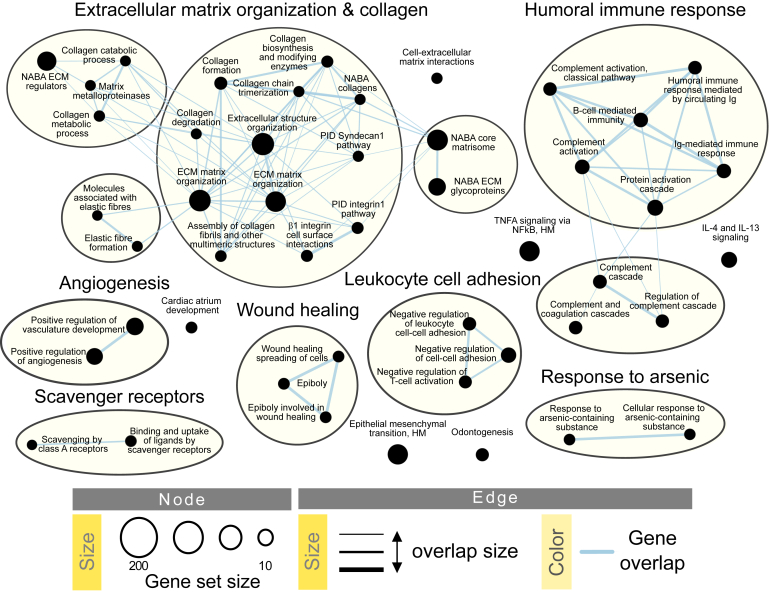
**CC differs from UC in gene pathways related to leukocyte adhesion and the humoral immune response, angiogenesis, wound healing, and extracellular matrix (ECM).** Enrichment map of gene expression in which nodes represent UC phenotype pathways (normalized enriched scores) created with FDR *Q* value <0.05, and combined coefficient >0.375 with combined constant = 0.5. Unless otherwise stated, gene pathways were retrieved from Gene Ontology Biological Process database. The analysis did not result in any pathway enriched for auCC phenotype. Based on results from 9 auCC and 4 active UC patients.

To further explore the idea that CC displays common features with UC, we compared the messenger RNA expression of active CC with UC samples (Table 1, Figure 3). When compared with active CC samples, UC samples differed in the expression of 600 protein-coding genes. In particular, UC mucosal gene expression was enriched for extracellular organization and collagen-related genes, humoral immune response, angiogenesis, wound healing, and leukocyte cell adhesion processes when compared with auCC mucosa (Figure 3). To identify which genes are specifically involved in CC but not in UC pathogenesis, we compared the lists of DEGs between either auCC or UC samples and healthy control subjects, and filtered for those with absolute log_2_ fold changes above 2. The 161 resulting CC-specific genes were mostly involved in the metabolism of fatty acids and prostaglandins, and in peroxisome proliferator-activated receptor signaling pathways (Table 2; Supplementary Table 2).

**Table 2:** Enriched Gene Pathways From CC-Specific Differentially Expressed Genes

Database	Term	Adjusted *P* Value	Odds Ratio	Combined Score	Genes
GO Biological process	cellular protein complex localization (GO:0034629)	.2483	20.9082	154.6912	MIOS;NACC2;KLHL21
GO Biological process	protein complex localization (GO:0031503)	.2483	19.8068	143.7553	MIOS;NACC2;IFT46
GO Biological process	prostanoid metabolic process (GO:0006692)	.3624	24.9421	137.5235	HPGD;ACOX1
GO Biological process	regulation of monooxygenase activity (GO:0032768)	.2483	18.8155	134.0420	DDAH2;CALM1;CYGB
GO Biological process	alpha-linolenic acid metabolic process (GO:0036109)	.3624	22.6735	121.3471	FADS2;ACOX1
GO Biological process	prostaglandin metabolic process (GO:0006693)	.2550	17.1033	117.5756	EDN2;HPGD;ACOX1
GO Biological process	unsaturated fatty acid metabolic process (GO:0033559)	.2483	12.6108	97.8342	FADS2;SCD;ACOX1;MGLL
GO Biological process	unsaturated fatty acid biosynthetic process (GO:0006636)	.3624	19.1834	97.1681	HPGD;SCD
GO Biological process	peptidyl-threonine dephosphorylation (GO:0035970)	.3624	17.8122	87.9383	PPM1A;DUSP10
GO Cellular Components	DNA-directed RNA polymerase II, core complex (GO:0005665)	.3937	16.6239	80.0785	URI1;POLR2D
KEGG	PPAR signaling pathway	.0046	11.2548	117.5080	RXRB;FADS2;GK;ACOX1;SCD;AQP7
KEGG	Biosynthesis of unsaturated fatty acids	.1033	15.6764	104.1832	FADS2;SCD;ACOX1
WikiPathways	Estrogen Receptor Pathway WP2881	.1777	22.6735	121.3471	ACOX1;PDK4
WikiPathways	Sulindac Metabolic Pathway WP2542	.3563	30.9922	100.0630	MSRA
WikiPathways	ID signaling pathway WP53	.2153	17.8122	87.9383	PAX8;ID3
WikiPathways	PPAR signaling pathway WP3942	.0308	10.2238	86.8204	RXRB;FADS2;ACOX1;SCD;AQP7

GO, Gene Ontology; KEGG, Kyoto Encyclopedia of Genes and Genomes; PPAR, peroxisome proliferator-activated receptor.

### Budesonide Modulates CC Transcriptional Program in Responsive Patients

**Figure 4:**
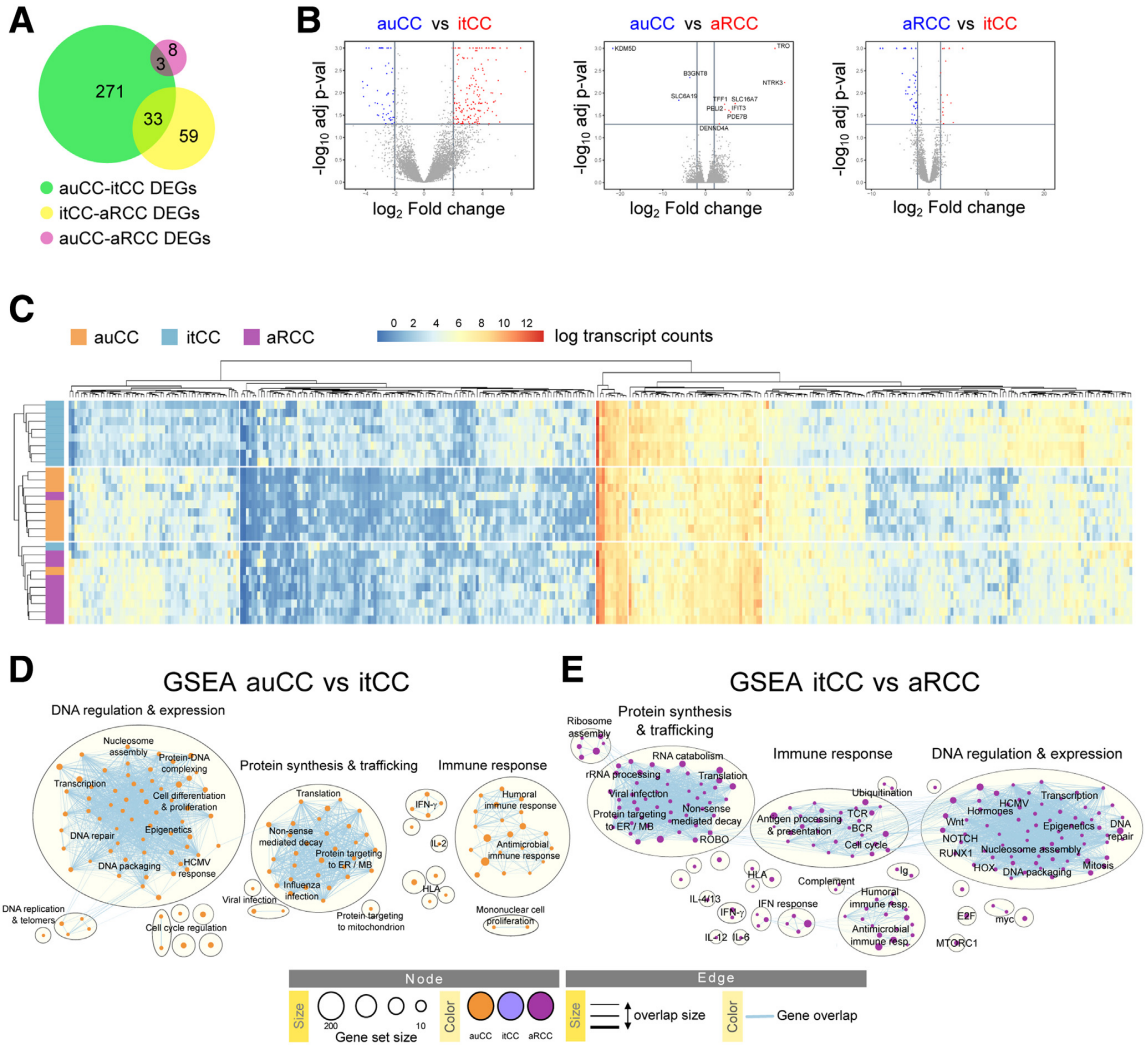
**Budesonide treatment dampens cell proliferation and the immune response but not in steroid-resistant CC patients.** (*A*) Diagram displaying the numbers of DEGs coding for proteins between CC patient subgroups. The expression of 271 genes changed between auCC and itCC samples (green), while 59 between itCC and aRCC samples (yellow), and 8 between auCC and aRCC (pink). A total of 36 genes changed between different CC subgroups. (*B*) Volcano plots showing differentially regulated protein-coding genes in auCC compared with itCC (left), and to aRCC (center) samples, and between itCC compared with aRCC samples (right). Red and blue colors indicate the group whose genes are upregulated in. (*C*) Heatmap showing normalized log_2_-transformed fold changes (regularized log function in R) of RNA-seq transcript counts from all the differentially expressed protein-coding genes between CC subgroups in comparison to healthy control samples. Heatmap rows and columns are split according to hierarchical clustering. (*D*, *E*) Enrichment maps from GSEA performed including all DEGs (*E*) between auCC and itCC and (*E*) between itCC and aRCC, ranked based on adjusted *P* values. GSEA for the comparison of active CC samples (untreated vs refractory) did not reveal any enriched pathway (not shown). n = 9–13 samples per group.

Budesonide is the only effective treatment for CC,^1^^,^^2^^,^^15^ but its effects in colitis are not fully understood. We therefore analyzed the transcriptomic profile of active CC patients that volunteered for extra biopsy sampling after achieving clinical remission following budesonide treatment after an average period of 6 weeks (inactive/treated CC [itCC]), and another set of patients who, despite this therapy, never responded to treatment and still suffered from watery diarrhea after 12 weeks (active/refractory CC [aRCC]). Of note, budesonide did not seem to affect the thickness of the collagenous band in most of our patients, independently of the response outcome (Table 1; Figure 1*A*). RNA sequencing (RNA-seq) analysis of the 9 CC samples obtained after successful treatment (itCC) revealed that 307 protein-coding genes were differentially expressed (DEGs) compared with the matched active disease samples (auCC samples) (Figure 4*A* and *B*). In contrast, 92 genes differed between steroid-responsive and nonresponsive patients (itCC vs aRCC) (Figure 4*B* and *C*). Based on the expression of all these genes and in comparison with healthy control samples, active disease samples clustered together and displayed very similar expression profiles (Figure 4*C*). Genes that were associated with active CC forms contributed to DNA regulation and expression, protein synthesis, and trafficking, and to immune responses, as highlighted by GSEAs of itCC samples compared with auCC or aRCC samples (Figure 4*D* and *E*; Supplementary Tables 3 and 4). Of note, of the 161 CC-specific genes identified previously, the expression of 149 was normalized after treatment with budesonide, as observed when itCC data were compared with healthy control subjects (Supplementary Table 2).

**Figure 5:**
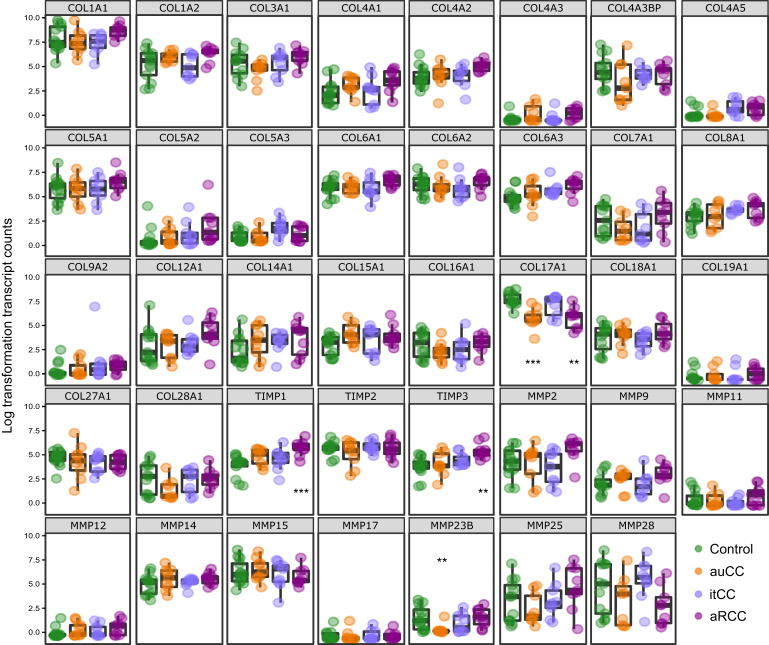
**Gene expression of collagen and ECM-related genes in CC mucosa.** Normalized log_2_-transformed fold changes (using the regularized log function in R) of RNA-seq transcript counts. Healthy control subjects (Hc) are shown in green, auCC samples in orange, inactive/treated CC (itCC) samples in blue, and active/refractory CC (aRCC) samples in purple. n = 9–13 samples per group. Statistically significant differences relative to Hc samples are shown as ∗∗*P <* .01, and ∗∗∗*P <* .001.

Because the main CC histological feature is a thick collagenous band, we explored the expression of extracellular matrix components, including collagen, matrix metalloproteinases (MMPs), and MMP inhibitors (tissue inhibitors of metalloproteinases [TIMPs]). Of collagens, we not only failed to detect an increase in gene expression but identified a decrease of the *COL17A1* gene in active CC forms, which encodes for collagen type XVII α1 chain (Figure 5). In addition, we detected an increase in the expression of TIMPs 1 and 3, mostly in aRCC samples (Figure 5).

**Figure 6:**
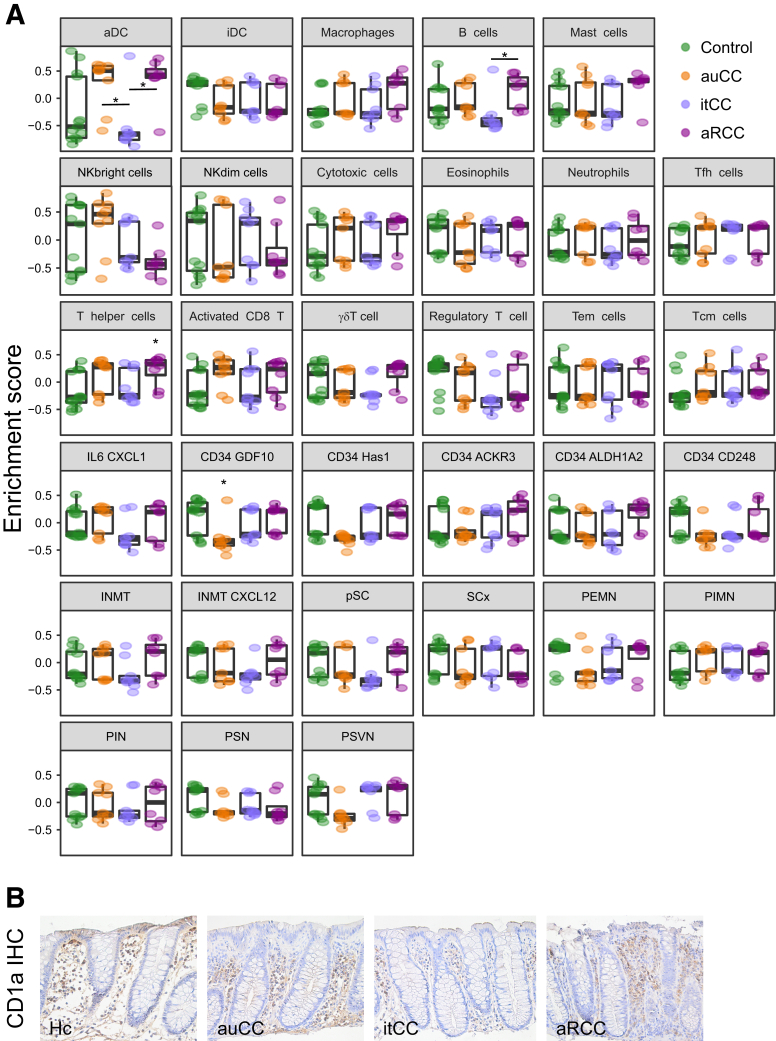
**Immune and stroma cell types in CC mucosa.** (*A*) GSVA computed for all different immune and stroma cell types from RNA-seq data displaying enrichment scores (median with interquartile range). Healthy control subjects (Hc) are shown in green, auCC samples in orange, inactive/treated CC (itCC) samples in blue, and active/refractory CC (aRCC) samples in purple. (*B*) Representative immunohistochemistry (IHC) images of CD1a staining in paraffin-embedded sections from Hc, auCC, itCC, and aRCC colonic samples. Note the brown staining in active CC forms is stronger than in Hc subjects due to an increased cellular infiltration in the stroma. Fibroblastic stroma cells are subdivided into inflammatory and chemokine-producing cells (IL6 CXCL1), CD34-derived cells, INMT^+^ cells, proliferative stroma cells (pSC), and other stroma cells (SCx); enteric neurons are subdivided into putative excitatory motor neurons (PEMN), putative inhibitory motor neurons (PIMN), putative interneurons (PIN), putative sensory neurons (PSN), and putative secretomotor/vasodilator neurons (PSVN). n = 9–13 samples per group. Statistically significant differences relative to Hc samples are shown as ∗*P <* .05, unless other comparison is indicated. activated CD8, activated CD8^+^ T cells; aDC, activated dendritic cells; iDC, immature dendritic cells; NK, natural killer; regulatory T, regulatory T cells; Tcm cells, central memory T cells; Tem cells, effector memory T cells; Tfh cells, follicular T helper cells; γδT cells, TCRγδ^+^ T cells.

To define the profile of cells present in the mucosa of each CC patient sample group, we computed gene set variation analysis (GSVA) of stroma and immune populations as previously described (Figure 6*A*; Supplementary Table 5).^16^ We particularly noticed an apparent decrease of CD34^+^ GDF10^+^ stroma cell profile in auCC samples (Figure 6*A*). In contrast, immune cells such as active dendritic cell and T helper cell profiles were estimated to be increased in active CC, especially in steroid-refractory mucosa (Figure 6*A*). The dendritic cell infiltration in CC mucosa was confirmed by immunohistochemistry staining for CD1a (Figure 6*B*). Enteric neuron and innate immune response cells did not change (Figure 6*A*).

**Figure 7:**
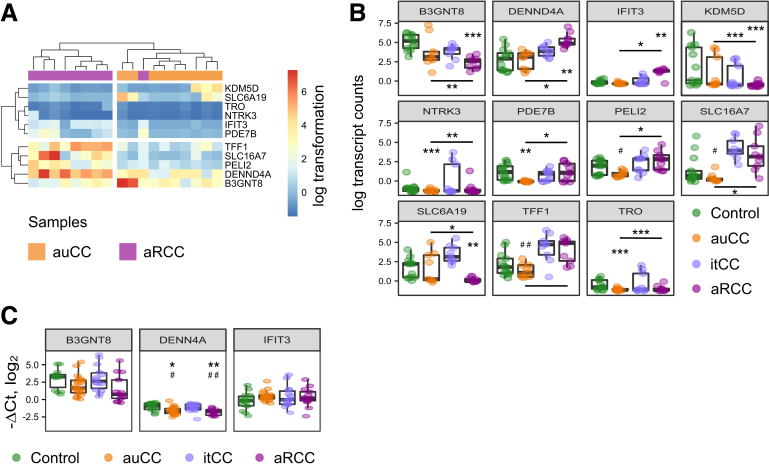
**DEGs between auCC and aRCC colonic mucosa.** (*A*, *B*) Normalized log_2_-transformed fold changes (using the regularized log function in R) of RNA-seq transcript counts (log TC) from DEGs between auCC and aRCC CC mucosa (*A*) displayed as a heatmap or (*B*) as detailed individual plots including samples from Hc subjects and all CC groups (median with interquartile range). Selected genes for further RT-qPCR validation are highlighted in red. (*C*) Log_2_ fold changes (–ΔCt values) (median with interquartile range) in gene expression of *B3GNT8*, *DENN4A*, and *IFIT3* analyzed by quantitative PCR. *HPRT1* was used as a housekeeping control. Hc subjects are shown in green, auCC samples in orange, itCC samples in blue, and aRCC samples in purple. n = 9–13 samples per group for RNA-seq analyses; n = 13–20 samples per group for RT-qPCR validation. Statistically significant differences relative to Hc samples are shown as ∗*P <* .05, ∗∗*P <* .01, and ∗∗∗*P <* .001, unless other comparison is indicated; statistically significant differences relative to itCC samples are shown as #*P <* .05, and ##*P <* .01.

Of the 11 DEGs identified between auCC and aRCC samples, we opted to validate by reverse-transcription quantitative polymerase chain reaction (RT-qPCR) the 3 genes with higher fold change between these 2 groups using an extended CC patient cohort (Figure 7; Table 3). However, none of them resulted statistically different in auCC when compared with aRCC samples (Figure 7).

**Table 3:** Clinical and Demographic Characteristics of CC Patient Validation Cohorts and Control Subjects Included in RT-qPCR

Variable	RT-qPCR With All Groups				RT-qPCR of itCC Samples: Relapse vs No Relapse		
	Hc	auCC	itCC^a^	aRCC	No Relapse (Biopsies and Blood)	Relapse (Biopsies)	Relapse (Blood)
Total number of subjects	14	20	14	13	6	8	10
Number of subjects included in RNA-seq	8	3	6	7	4	2	5
On budesonide treatment	No	No	Yes	No	Yes	Yes	Yes
Steroid responders	—	Yes	Yes	No	Yes	Yes	Yes
Female, %	42.86	75	78.57	92.31	83.33	75	70
Age, y	62 (60–71)	63 (28–86)	66 (35–86)	54 (25–75)	73 (49–86)	61 (35–76)	51 (27–73)
Stools/day	—	6.90 (3–12)	1.43 (1–2)	8.92 (4–15)	1.33 (1–2)	1.50 (1–2)	1.20 (1–2)
Watery stools/day	—	6.70 (2–12)	0 (N/A)	8.38 (4–15)	0 (N/A)	0 (N/A)	0 (N/A)
Collagenous band, μm	—	32.15 (12–52)	29.57 (2–72)	33.38 (10–68)	30.50 (5–72)	28.88 (2–50)	22.00 (2–45)

NOTE. Values are n or mean (range), unless otherwise indicated.

auCC, active/untreated collagenous colitis; aRCC, active/steroid-refractory collagenous colitis; CC, collagenous colitis; Hc, healthy control subjects; itCC, inactive/treated collagenous colitis; N/A, not applicable; RNA-seq, RNA sequencing; RT-qPCR, reverse-transcription quantitative polymerase chain reaction.

a Matched samples from itCC patients were collected before and during treatment with budesonide. Note that samples before treatment (active disease) were included in the group of auCC samples, whereas samples during treatment were included as itCC samples.

Taken together, GSEA and GSVA indicate that budesonide affects the immune response in CC by decreasing the protein trafficking and antigen presentation in cells, and possibly decreasing the number of active antigen-presenting cells, but only in steroid-responsive patients. To note, active CC forms (ie, naïve untreated CC and budesonide-refractory CC) do not significantly differ at the transcriptomic level.

### Budesonide Fails to Completely Restore the Expression of Dysregulated Immune-Related Genes in Responding CC Patients

**Figure 8:**
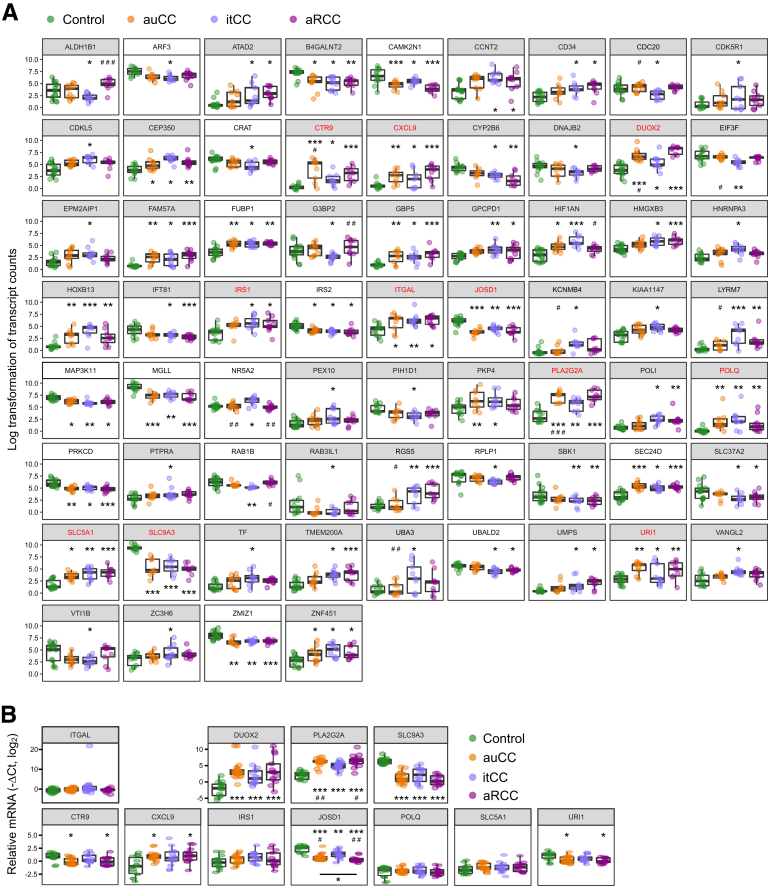
**DEGs between Hc subjects and itCC colonic mucosa.** (*A*) Normalized log_2_-transformed fold changes (regularized log function in R) of RNA-seq transcript counts from differentially expressed genes between Hc subjects and itCC mucosa (median with interquartile range). Selected genes for further RT-qPCR validation are highlighted in red. Genes with white background had an absolute log_2_ fold change value <2 between Hc and itCC samples. (*B*) Log_2_ fold changes (–ΔCt values) (median with interquartile range) in gene expression of selected genes analyzed by quantitative RT-qPCR. *HPRT1* was used as a housekeeping control. In both panels, Hc are shown in green, auCC samples in orange, itCC samples in blue, and aRCC samples in purple. n = 9–13 samples per group for RNA-seq analyses; n = 13–20 samples per group for RT-qPCR validation. Statistically significant differences relative to Hc samples are shown as ∗*P <* .05, ∗∗*P <* .01, and ∗∗∗*P <* .001, unless other comparison is indicated; statistically significant differences relative to itCC samples are shown as #*P <* .05, and ##*P <* .01.

Budesonide efficiently maintains clinical remission in 61%–77% of the patients during long-term treatment, but remission continues only in approximately 20% when it is discontinued. To explore possible underlying pathomechanisms, we first aimed to identify CC-associated DEGs that are unaffected by steroid treatment and may thus contribute to the reoccurrence of symptoms. To this end, we analyzed DEGs between healthy control and itCC samples, and selected the genes with higher fold changes between these 2 groups and similar expression pattern in itCC and active CC samples for subsequent validation (Figure 8*A*). Of the 11 selected genes, qPCR analyses confirmed that several genes related to immune response (*DUOX2*, *PLA2G2A*, *CXCL9*), DNA transcription and protein ubiquitination (*CTR9*, *JOSD1*, *URI1*), and ion transport (*SLC9A3*) were not restored to normal levels after budesonide treatment (Figure 8*B*; Table 3).

**Figure 9:**
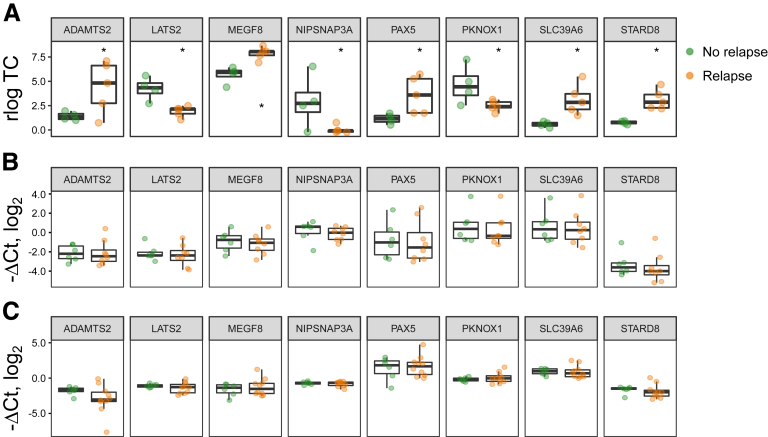
**DEGs in itCC samples from patients that experienced a disease relapse against those who did not.** (*A*) Normalized log_2_-transformed fold changes (using the regularized log function in R) of RNA-seq transcript counts (log TC) (median with interquartile range) of DEGs between itCC samples from patients that experienced a disease relapse (orange), or did not (green). n = 4–5 samples per group. (*B*, *C*) Log_2_ fold changes (–ΔCt values, median with interquartile range) in gene expression from DEGs identified by RNA-seq in (*B*) colonic samples or (*C*) blood of itCC patients that suffered from a disease relapse (orange), or did not (green), as analyzed by RT-qPCR. *HPRT1* was used as a housekeeping control. n = 6–10 samples per group for validation analyses. All primers detect all coding transcript variants of the indicated gene. n = 4–5 samples per group. Statistically significant differences are shown as ∗*P <* .05.

Next, we asked if any DEGs could have predictive value for disease relapse. For this, we separated itCC samples included in our RNA-seq analysis into patients that suffered from a disease relapse or not in the following months and found 8 DEGs (Figure 9*A*; Table 3). However, we were not able to identify any of them as potential CC relapse biomarkers b RT-qPCR analyses did not show significant changes in gene expression either in biopsy or in peripheral blood samples in the validation cohort (Figure 9*B* and *C*; Table 3).

In summary, genes that remain dysregulated despite treatment with budesonide could be targets for new therapies for CC patients, and validation in alternative patient cohorts could be of interest. In contrast, we failed to find a biomarker that predicts CC relapse when patients are under budesonide treatment.

### The Intestinal Epithelial Cell Transcriptome Is Altered in CC

**Figure 10:**
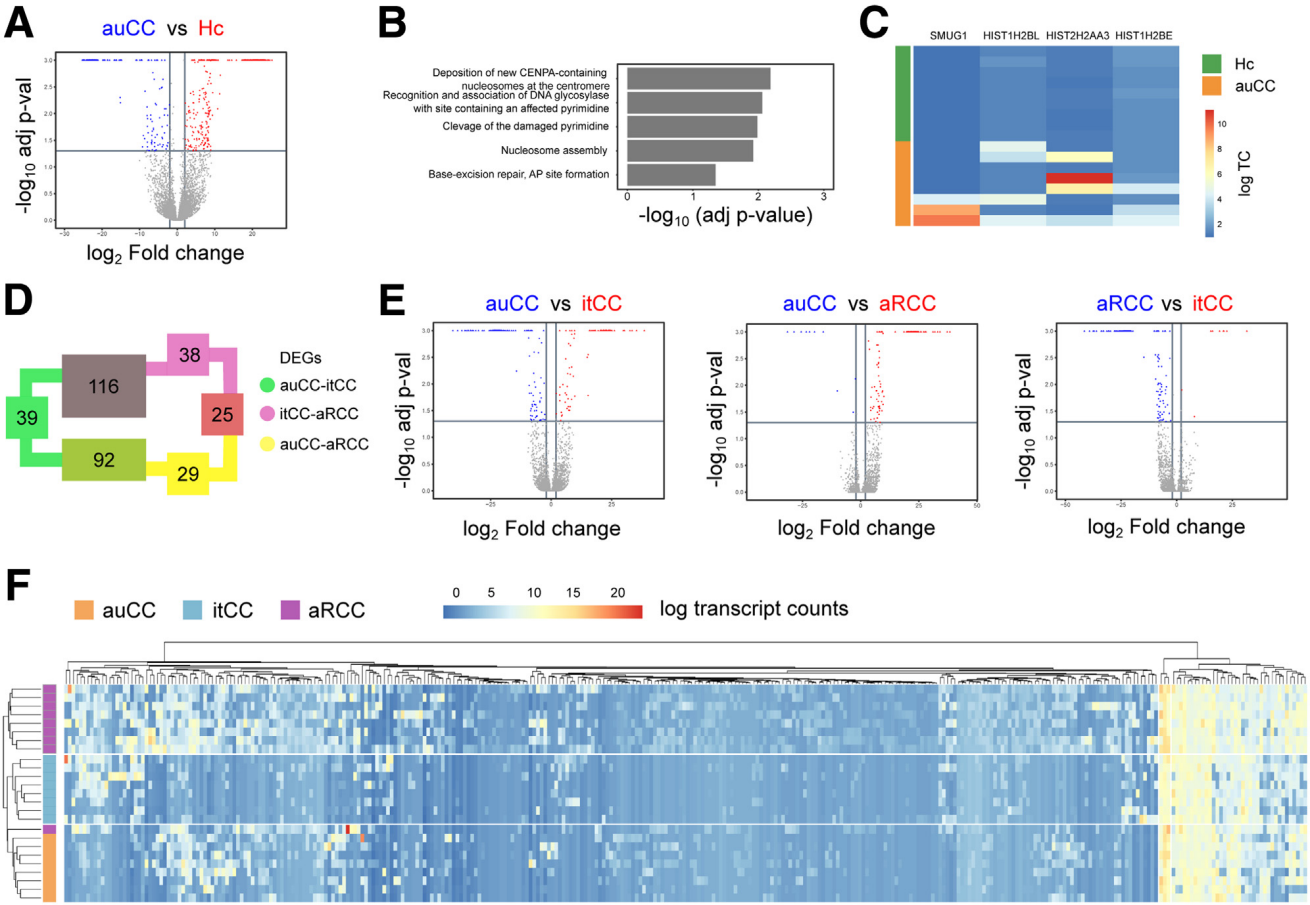
**CC intestinal epithelial cell gene expression compared with Hc subjects.** (*A*) Volcano plot showing differentially expressed protein-coding genes in auCC (upregulated genes in blue) compared with Hc subjects (upregulated genes in red). (*B*) GSEA performed including DEGs between auCC and Hc samples, ranked based on adjusted *P* values. (*C*) Heatmap showing normalized log_2_-transformed fold changes (using the regularized log function in R) of RNA-seq transcript counts (log TC) from leading genes contributing to the enriched gene pathways shown in *B*. (*D*) Diagram displaying the numbers of DEGs coding for proteins between CC patient subgroups. The expression of 39 genes changed between auCC and itCC samples (green), while 29 did between auCC and aRCC samples (yellow), and 38 did between itCC and aRCC samples (pink). A total of 339 genes changed between different CC subgroups. (*E*) Volcano plots showing differentially regulated protein-coding genes in auCC compared with itCC samples (left) or to aRCC samples (center), and between itCC compared with aRCC samples (right). Red and blue colors indicate the group were genes are upregulated. (*F*) Heatmap showing normalized log_2_-transformed fold changes (regularized log function in R) of RNA-seq transcript counts (log TC) from all the differentially expressed protein-coding genes between CC subgroups in comparison with Hc samples. Heatmap rows and columns are split according to hierarchical clustering. Hc subjects are shown in green, auCC samples in orange, itCC samples in blue, and aRCC samples in purple.

Despite the mucosal immune activity in CC, the mucosa is macroscopically intact.^1^ Synergistic electrolyte and water transport imbalance as well as nuclear factor κB activation implicates involvement of intestinal epithelial cells (IECs) in CC pathogenesis.^9^, ^10^, ^11^, ^12^ Thus, we microdissected intestinal epithelia from paraffin-embedded tissue sections from samples of our initial patient cohort (Table 1) to explore the role of IECs in CC pathogenesis by RNA-seq. Comparison of active untreated CC samples with healthy control subjects showed only 5 enriched pathways, which belonged to DNA organization in the cell nuclei, with leading genes involved in chromatin remodeling and repair events (Figure 10*A–C*). The amount of differentially expressed genes between CC subgroups was similar as in bulk biopsies (374 vs 339 DEGs) (Figure 10*D* and *E*) and, surprisingly, the matched samples taken before and after budesonide treatment of responsive patients clustered together (Figure 10*F*).

**Figure 11:**
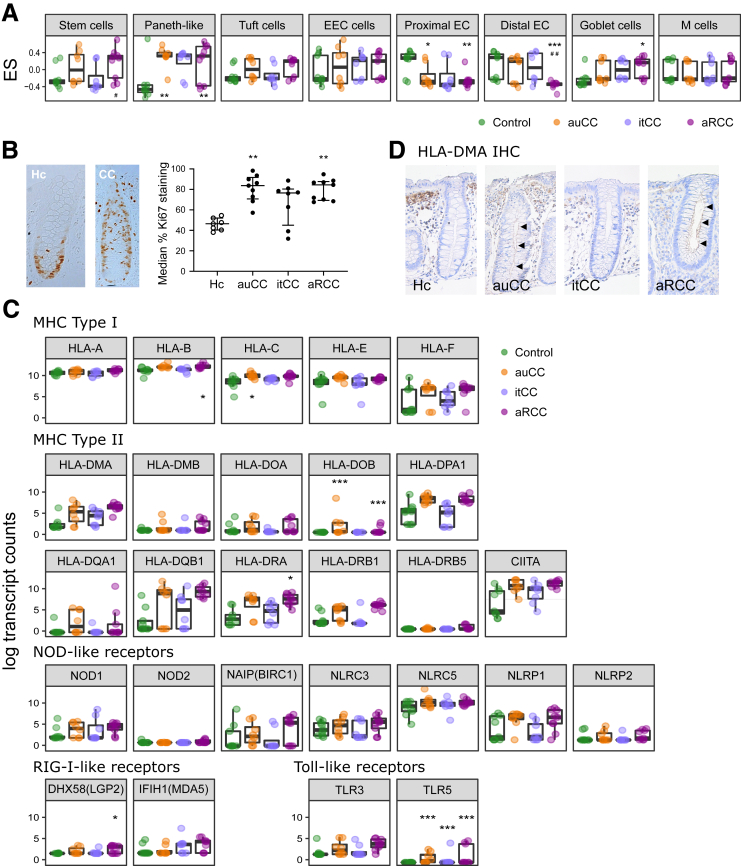
**CC IECs’ transcriptional profile suggest cell proliferation and recognition of antigens better than IEC from control subjects.** (*A*) GSVA computed for different epithelial cell types from RNA-seq data displaying enrichment scores (ES) (median with interquartile range). Hc subjects are shown in green, auCC samples in orange, itCC samples in blue, and aRCC samples in purple. (*B*) Representative IHC images of longitudinally sectioned epithelial glands stained for Ki67 proliferation marker (brown) in paraffin-embedded sections of Hc and CC colonic mucosa (left). Analysis of Ki67 relative staining to total crypt length is shown on the right (median with interquartile range, median of 9 crypts/patient). (*C*) Pattern recognition receptor and *HLA* gene expression in intestinal epithelial cells from CC mucosa as normalized log_2_-transformed fold changes (using the regularized log function in R) of RNA-seq transcript counts (median with interquartile range) of pattern recognition receptors including members from the NOD-like, RIG-I-like, and Toll-like families, and *HLA* genes (divided into genes coding for major histocompatibility complex [MHC] type I and II proteins). Group colors are the same as in *A*. (*D*) Representative IHC images of HLA-DMA staining of paraffin-embedded sections from Hc, auCC, itCC, and aRCC colonic samples. Note the brown staining in the apical side of IEC sin crypts from active samples (black arrows). n = 7–13 samples per group. Statistically significant differences relative to Hc samples are shown as ∗*P <* .05, ∗∗*P <* .01, and ∗∗∗*P <* .001; statistically significant differences relative to itCC samples are shown as #*P <* .05 and ##*P <* .01. EEC cells, enteroendocrine cells; EC, enterocytes; M cells, microfold cells.

To identify key cellular components, we computed GSVA of 8 IEC populations adapted from the intestinal epithelial atlas produced by Haber et al*.*^17^ The enrichment scores of stem cells and Paneth-like cells in active CC forms suggested an increased activity within colonic crypts during the disease (Figure 11*A*; Supplementary Table 5). However, staining with the proliferation marker Ki67 corroborates an increased proliferation in active CC colonic crypts that is not fully restored by budesonide (Figure 11*B*). Interestingly, the enterocyte profile is decreased in CC, especially enterocytes from the proximal intestine, but this result should be interpreted carefully due to the extrapolation of these profiles from the small intestine mouse atlas (Figure 11*A*; Supplementary Table 5).

Because some IECs can contribute to immune responses (eg, Paneth-like and goblet cells),^18^ we explored the expression of pattern recognition receptors and *HLA* genes in IEC (Figure 11*C*). Changes in pattern recognition receptors, including NOD-like receptors, accounted for very modest, nonsignificant alterations within all CC subgroups. Moreover, *HLA*-related genes were prone to increase in active CC samples, which was confirmed by increased HLA-DMA protein levels in the apical side of IECs from active CC samples (Figure 11*C* and *D*) Altogether, these data show that CC mucosal transcriptome is altered and that epithelial cells might contribute toward immune responses and disease pathogenesis in CC.

### aRCC Shares Similarities With UC

**Figure 12:**
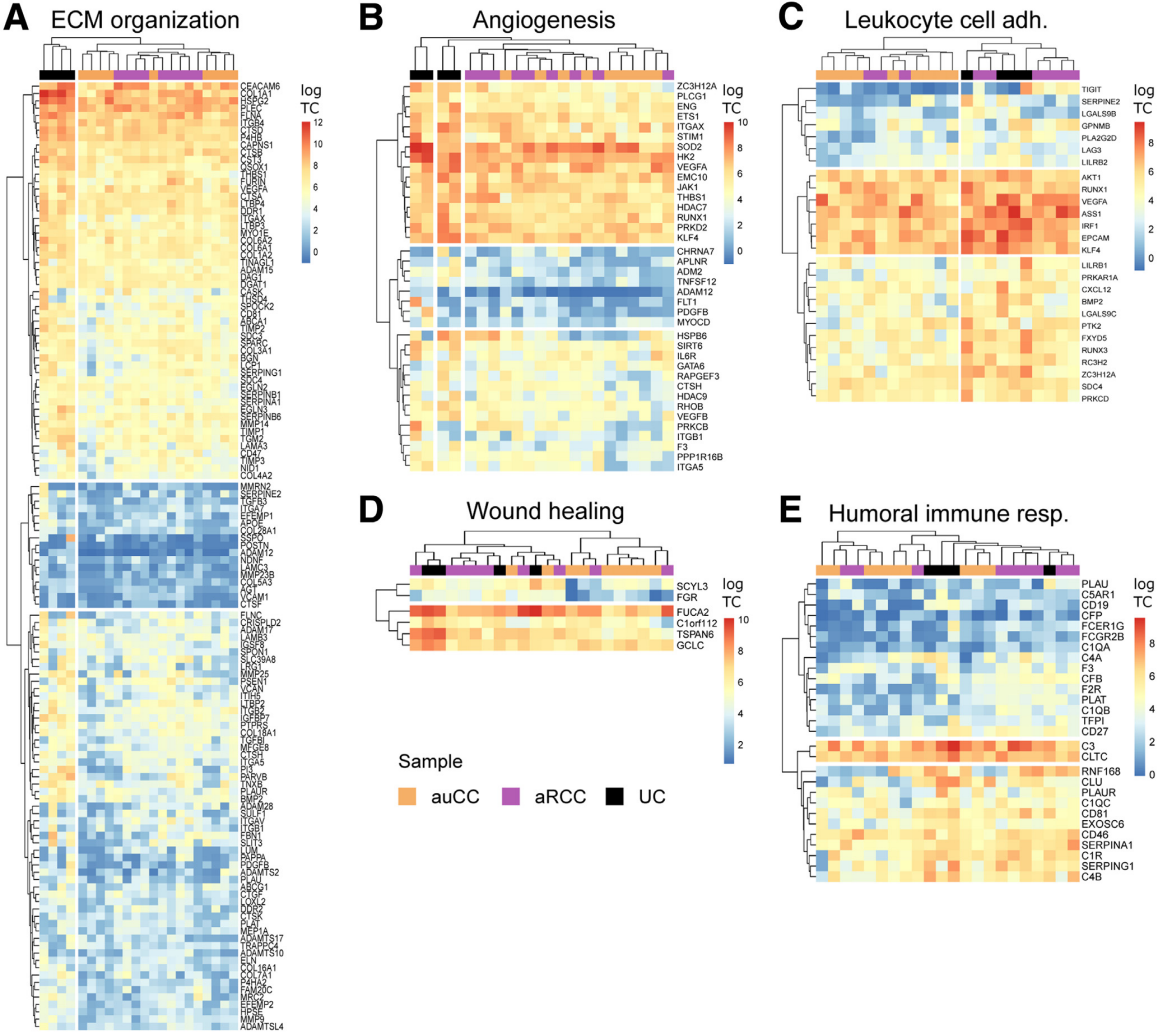
**Active refractory CC mucosa shares similarities with ulcerative colitis.** (*A–E*) Heatmaps showing regularized log_2_-transformed fold changes of RNA-seq transcript counts from leading genes contributing to the enriched gene pathways in UC, auCC and aRCC colonic mucosa related to ECM organization and (*A*) collagen, (*B*) angiogenesis, (*C*) leukocyte cell adhesion, (*D*) wound healing, and (*E*) and humoral immune response. auCC samples are shown in orange, aRCC samples in purple, and UC samples in black. Heatmap rows and columns are split according to hierarchical clustering. n = 4–9 samples per group.

As mentioned previously, CC displays common features with UC (Figure 3), but refractory CC has never been compared with UC. Our GSEA between aRCC and UC samples showed that only the different gene pathway was that regulating vasoconstriction, with *ADM* and *TRPM4* as leading genes (data not shown). When major pathways identified after auCC–UC comparison (from Figure 3) were explored in detail (Figure 12), UC samples segregated from CC samples for genes related to extracellular matrix organization and angiogenesis (Figure 12*A* and *B*). Interestingly, leukocyte cell adhesion and wound healing gene pathways clustered UC together with aRCC samples (Figure 12*C* and *D*). Therefore, in this scenario, the transcriptional landscape of steroid-refractory CC patients appears similar to UC, which indicates that UC treatments focused on preventing immune cell adhesion or promoting wound healing processes might be useful for treating aRCC.

## Discussion

Because the etiology and pathobiology of CC remains poorly understood, here we describe a transcriptional alteration of genes related to antigen presentation, lipopolysaccharide response, and IFN signaling routes, which might point to a role for Gram-negative bacteria and viruses in CC pathogenesis. Dense genotyping of immune-related loci in 10.13039/100010309CC identified HLA-DQ2 as a genetically predisposing factor in CC,^1^^,^^7^ which supports our findings. It has also been suggested that an abnormal translocation of bacteria could trigger the inflammation in CC, and thus attract immune cells into the mucosa.^1^^,^^19^ Microbiota studies so far have identified a decreased concentration of the epithelial-protective *Akkermansia muciniphila* bacteria (Gram-negative) and the Clostridia-related, butyrate-producing Ruminococcaceae bacteria family (Gram-positive).^20^^,^^21^ Especially a decreased abundance of Ruminococcaceae is, in general, associated with loose stools and is restored after treatment with budesonide, hence the bacterial microbiota might be affected by the lumen content flow.^20^^,^^22^ This profile is shared with other IBD forms,^20^^,^^22^ but whether dysbiosis is causative or consequential to the inflammation remains unknown. Interestingly, the translocation of chemically killed *Escherichia coli* K12 (Gram-negative) is increased in Ussing chamber mucosal barrier assays using CC biopsy samples,^19^ and fecal stream diversion has been effective treating nonresponsive CC patients.^23^^,^^24^ On the other side, Epstein-Barr virus has been detected in CC mucosa with even higher DNA detection than the levels reported in UC.^25^ Because this and other herpes viruses have also been found to be associated with IBD clinical morbidity,^26^ it would be worth to explore their presence in CC in depth. In addition, we did not find evidence of autoimmune mechanisms, and findings of some autoantibodies have only been reported in small studies and do not correlate with clinical symptoms.^27^, ^28^, ^29^ Altogether, owing to the similarities with classical IBD forms,^30^ CC can thus be described as an IBD in which mucosal cells overreact against microbiota of diverse nature. Still, we have proposed 161 DEGs that could specifically contribute to CC pathomechanisms, including genes related to fatty acid and prostaglandin metabolism, and peroxisome proliferator-activated receptor signaling. To note, these will require further validation in additional cohorts with larger inflammatory control subsets because the number of UC samples included in this study was limited.

Dendritic cells are professional antigen-presenting cells capable to determine the fate of antigen-specific immune outcomes. In the healthy gut, dendritic cells promote immune tolerance toward nutrients and commensals through regulatory T cell and IgA-producing B cell responses.^31^ Among the infiltrating cells in CC mucosa, we identified an increase in genes associated with active dendritic cells and an increased infiltration of CD1a^+^ cells. Similarly, IBD inflamed mucosa displays increased numbers of these cells that overexpress pattern recognition receptors, hence promoting a proinflammatory Th1/Th17 response.^31^ Usually, dendritic cells rapidly respond to microenvironment changes, such as signals from IECs, and promote a tolerogenic immune response.^32^^,^^33^ However, debilitated IEC tight-junctions, as reported in IBDs, facilitate the passage of invading microorganisms that induce a proinflammatory response.^34^, ^35^, ^36^ In particular, HLA expression in IECs is responsible for Crohn’s disease and UC IEC ability to induce CD4^+^ T cell proliferation and IFNγ secretion.^37^ Therefore, CC IECs might exert similar effects through HLA-DMA. In addition, we and others reported an increased IEC proliferation in CC colonic crypts, an increased lysozyme expression, and a possible increase in antigen presentation that, in turn, could disrupt IEC homeostasis and their communication with the underlying stroma cells.^34^^,^^38^^,^^39^ Indeed, we also report here an increased expression of metalloprotease inhibitors *TIMP1* and *TIMP3*, which would prevent collagen degradation. Madisch et al^40^ also associated CC with a genetic variation in the *MMP-9* gene and, in our results, we can observe a tendency for MMP-9 messenger RNA upregulation in active CC that is corrected in budesonide-responding CC patients (Figure 5). In addition, we report a potential dysregulation of a subset of CD34^+^ GDF10^+^ stroma cells identified in a single-cell RNA-seq mouse atlas that would worth validation. Whether these cells could also be responsible for decreased *COL17A1* expression remains unknown and would require a comprehensive analysis of fibroblast—IEC interactions.

Despite identification of active antigen-presenting cells in CC mucosa and increased expression of chemoattractant genes for leukocyte populations (eg, *CXCL9*), we and others have not found evidence of increase of T cells (ie, CD4^+^ T helper, CD8^+^ cytotoxic T cells, or T regulatory cells).^41^^,^^42^ However, our results are limited to the use of gene expression data and the power of GSVA algorithm to estimate cellular population frequencies and due to the extrapolation of mouse gene identifiers to human tissue. Conversely, Kumawat et al^38^^,^^43^ found evidence of increased T cell numbers in CC mucosa, identifying the disease as a mixed Th17/Tc17 and Th1/Tc1 IBD using flow cytometry, which is a more reliable approach. Still, Kumawat et al’s results could not be replicated in the cohort analyzed by Carrasco et al,^41^ where they only found an increase in CD3^+^CD4^–^CD8^–^ double negative (DN) T cells. Despite DN T cell abundance in CC and autoimmune disorders, the function of these cells is not well understood.^44^ They could be responsible of the increased expression of the pro-inflammatory cytokines found in CC mucosa, but DN T cells have also been attributed with anti-inflammatory properties.^41^^,^^45^^,^^46^ Whether the inflammation is effectively restrained by suppressor mechanisms in CC is not clear but will match with the lack of macroscopic mucosal damage^1^; hence, the exploration of the dendritic cell–IEC and the IEC–T cell interfaces might further clarify CC pathogenesis.

Budesonide is the only established therapy for CC but is an unspecific anti-inflammatory drug with ambiguous effects.^1^ Besides its anti-inflammatory properties, we observed a generalized decline in the expression of genes related to DNA regulation, protein synthesis and trafficking, and cell cycle regulation when CC patients responded to budesonide, which might be a secondary effect of the tissue restoration to normalcy as budesonide resolves the inflammation. Nonetheless, this inflammation is not completely abrogated because genes involved in innate immunity and cell recruitment remain increased after treatment (eg, *DUOX2*, *PLA2G2A*, and *CXCL9*). Thus, targeting of residual dysregulated genes could support low-dose budesonide therapy to ensure long-term clinical remission of CC patients. Despite our efforts to identify markers in budesonide-responding patients (itCC samples) that could indicate an upcoming disease relapse, we found that RNA-seq DEGs were false positive results after RT-qPCR corroboration, which supports that data validation with different techniques is mandatory when exploiting results for clinical application.

Steroid-refractory patients account for 10%–20% of CC individuals included in clinical trials and represent a clinical challenge.^47^, ^48^, ^49^, ^50^ In fact, no previous pathological characterization of aRCC has been reported to date. Despite the resemblance of aRCC transcriptome with auCC samples, we observed that leukocyte cell adhesion and wound healing processes are similarly altered in aRCC and UC but differ from auCC samples. This suggest that refractory CC could be a distinct disease entity with potentially unique pathomechanisms. In our previous work, we explored the restoration of water malabsorption in CC colon after budesonide treatment, and also found disparities between water channel aquaporin (AQP) 8 protein levels auCC and aRCC patients.^12^ Thus, supporting the hypothesis that despite similar to treatment-naïve CC, refractory CC behave differently and that these patients could benefit from alternative treatments, such as the ones available for UC patients. Indeed, immunomodulators and anti-tumor necrosis factor α therapies seem to have positive effects on aRCC patients.^1^^,^^51^^,^^52^ In addition, our results suggest that other biological treatments could be worth testing in aRCC. For instance, promising leukocyte/lymphocyte trafficking blocking therapies for UC patients under clinical trial testing include antibodies targeting α4 or β7 integrin subunits on leukocytes, or the mucosal addressin cell adhesion molecule MAdCAM-1 on endothelia, and modulators of the lymphocyte trafficking receptor sphingosine-1-phosphate (S1P).^53^ Actually, the already approved α4β7 blocking antibody vedolizumab induced clinical remission in almost half of the patients assessed by Rivière et al.^54^ Thus, novel IBD therapies would be worth to assess in large randomized clinical trials with refractory CC patients.

CC is emerging as a common disorder but, to date, no reliable disease-specific, noninvasive biomarker is available. However, we could propose gene targets that could be addressed for the development of novel therapies (eg, *DUOX2*, *PLA2G2A*, *CXCL9*, *CTR9*, *JOSD1*, *URI1*, and *SLC9A3*). Particularly, loss of function and excessive activity of enzymes producing reactive oxygen species DUOX2 and NOX1 have been suggested to contribute to gastrointestinal disease progression.^55^ Thus, NOX/DUOX inhibitors or reactive oxygen species inducers could be of use to develop novel therapies to treat CC. Similarly, other works found increased levels of proinflammatory cytokines and chemokines in CC^56^^,^^57^; hence, targeting the expression of genes related to inflammatory processes (eg, *PLA2G2A* and *CXCL9*) could prompt alternative treatment options.

In summary, this study provides a comprehensive landscape of CC pathology. Our results confirm that CC is an immune-mediated IBD in which luminal antigen presentation might occur via dendritic cells. Clinical remission can be achieved after budesonide treatment, but some genes remain dysregulated and may open the door for new treatments (eg, *DUOX2*, *PLA2G2A*, *CXCL9*). Still, budesonide-refractory CC could comprise a transcriptionally distinct disease entity, and owing to its similarities with UC, aRCC patients could benefit from treatments that are under investigation to treat UC.

## Materials and Methods

### Study Population

Biopsy samples from the descending colon were collected during scheduled colonoscopy in adult patients with CC patients at the Division of Gastroenterology at Linköping University Hospital, Sweden. CC was diagnosed according to the current guidelines,^2^ primarily clinical history and histopathological features, including a subepithelial collagen band of >10-μm thickness. Active CC was defined as more than 3 bowel movements per day or at least 1 watery bowel movement per day during a 1-week registration period. Clinical remission was defined as less than 3 bowel movements per day and no watery bowel movement within a 1-week period.^58^ A diagnosis of steroid-refractory CC was reached if patients did not achieve clinical remission after treatment for 12 weeks with 6- to 9-mg/d budesonide.^2^ Healthy volunteers were recruited from the local colon cancer screening program at Linköping University Hospital (Sweden) or St. Olav’s University Hospital (Norway); these individuals showed normal macro- and microscopic findings upon histopathological assessment, had normal bowel movements, and did not take any medication at the time of colonoscopy. We enrolled treatment-responsive patients with active CC, and some of them agreed to have additional biopsies and blood samples (collected in EDTA tubes; BD Biosciences, San Jose, CA) taken after reaching remission during budesonide treatment (after 6 weeks of treatment on average). We also obtained samples from steroid-refractory CC patients and healthy control subjects following the same bowel preparation procedure and biopsy taking from the descending colon as mentioned above. Active UC samples from patients without medication intake (n = 4) were used for comparison and collected at St. Olav’s University Hospital. These were diagnosed and assessed following the guidelines stated in the Mayo score system.^59^ Detailed patient characteristics can be found in Tables 1 (exploratory cohort) and 3 (validation cohort). Adjacent biopsy samples from the same mucosal area were stored in AllProtect (Qiagen, Hilden, Germany) or RNAlater (Thermo Fisher Scientific, Waltham, MA) for subsequent RNA extraction, or in phosphate-buffered saline (PBS) for fixation in paraformaldehyde, embedding in paraffin, and analyses using microscopy. Informed written consent was obtained from all subjects, and their data were handled according to current regulations (EU2016/679, corrigendum May 23, 2018). Ethical approval was issued by Linköping’s regional ethical committee to conduct studies in microscopic colitis, including CC (Dnr 2015/31-31), and by St. Olav’s University Hospital with approval from the Central Norway Regional Committee for Medical and Health Research Ethics no 2013/212/REKmidt.

### Genome-Wide Messenger RNA-Seq

Biopsies preserved in AllProtect or RNAlater (n = 13 healthy control subjects, n = 9 per CC group, and n = 4 UC) were homogenized in RLT buffer from RNeasy Mini Kit (Qiagen) supplemented with 1% 2-mercaptoethanol using a T10 Ultra Turrax homogenizer (IKA; Thermo Fisher Scientific). Total RNA from homogenized biopsy samples was isolated using RNeasy Mini Kit following the manufacturer’s instructions. RNA from laser capture microdissection material (n = 8–9 per group) was isolated with RNeasy FFPE kit (Qiagen, see the following sections). RNA integrity was assessed using an Agilent RNA 6000 Pico kit on a 2100 Bioanalyzer (Agilent Technologies, Santa Clara, CA). The DV200 value, representing the percentage of RNA fragments more than 200 nucleotides long, was used as a measure of RNA quality. The range of DV200 values was 30%–70%.^12^ RNA sequencing libraries were constructed with SENSE totalRNA with Ribo cop rRNA depletion (Lexogene, Vienna, Austria), and single-read sequenced for 75 cycles to a depth of 25 million base reads on a HiSeq4000 instrument (Illumina, San Diego, CA), according to the manufacturer’s recommendations. FASTQ files were generated using bcl2fastq software v2.18 (Illumina). Data was analyzed using the R Bioconductor software v3.5.1 (R Foundation for Statistical Computing, Vienna, Austria), including SARTools v1.6.6 and DESeq2 v1.22.1 packages.^60^, ^61^, ^62^ Reads were aligned to the Ensembl GRCh38 genome version, release 92. RNA-seq data are available at Gene Expression Omnibus under the accession number GSE159010 (https://www.ncbi.nlm.nih.gov/geo/query/acc.cgi?acc=GSE159010).

### RNA-Seq Data Analysis

Differential gene expression from RNA-seq data was determined with linear models using DESeq2 v1.22.1 and significance decided by Benjamini-Hochberg false discovery rate (FDR)–adjusted *P* values <.05. Principal component analysis was computed after making the data homoscedastic using R. GSEA of CC-specific genes was performed using EnrichR and taking into account the outcome from Gene Ontology, Kyoto Encyclopedia of Genes and Genomes, and WikiPathway databases. GSEA for comparisons of 2 sample groups was performed in GSEA^63^^,^^64^ v4.0.3 (Broad Institute, Cambridge, MA) using updated guidelines.^65^ DEGs were filtered for genes with <10 counts and ranked according to their differential gene expression log_10_-adjusted *P* value and sign of log fold change. Human gene set annotations from Gene Ontology, Reactome, and other databases were downloaded from Bader’s Lab website excluding those inferred from electronic annotations (https://download.baderlab.org/EM_Genesets/, release January 2020). Terms annotating more than 200 or <10 genes were discarded to improve biological interpretation. GSEA results were visualized using EnrichmentMap^66^ v3.2.0 and a FDR *P* value of <.05, a Jaccard Overlap Combined index of 0.375, and k constant of 0.5, and annotated using AutoAnnotate v1.3 applications for Cytoscape v3.7.2, and improved in Inkscape v0.92.4. Gene expression was normalized using the regularized logarithmic function in R for subsequent analyses and representation. Markers for stromal (fibroblasts and enteric neurons), immune and epithelial cell subtypes retrieved from publications (Supplementary Table 5)^16^^,^^17^^,^^67^^,^^68^ were used to compute GSVA using the GSVA^69^ package in R v3.6.3. Human orthologue annotations of epithelial and fibroblastic cell markers were procured from the Mouse Genome Database, Mouse Genome Informatics (http://www.informatics.jax.org; The Jackson Laboratory, Bar Harbor, ME), April 2020. Paneth-like cells were identified with a combination of Paneth-1 and Paneth-2 biomarkers^17^ and the inclusion of human Paneth cell–derived defensin genes.^70^ GSVA was analyzed using linear models using DESeq2 v1.22.1 and significance decided by Benjamin-Hochberg FDR adjusted *P* values <.05 using R software.

### Laser Capture Microdissection

Colonic biopsy samples collected in PBS were fixed in paraformaldehyde and embedded in paraffin (FFPE samples). Matched samples from 8 steroid-responsive CC patients before and during budesonide treatment, 9 steroid-refractory CC patients, and 9 healthy control subjects were used for microdissection. Laser capture microdissection was performed as previously described.^12^ Briefly, samples were cut into 10-μm sections and mounted on RNase-free MMI Membrane Slides (Molecular Machines and Industry; MMI AG, Eching, Germany); afterward, samples were stained with hematoxylin following standard protocols. The sections were dehydrated with 100% ethanol and xylene, followed by air-drying in a desiccator for at least 30 min. Intestinal epithelial cells (area of 10^6^ μm^2^, corresponding to approximately 10^4^ cells) were isolated from all samples with a UV-LCM MMI Cellcut device connected to an Olympus IX71 microscope (Olympus, Tokyo, Japan), and collected in MMI isolation caps with diffuser (all from MMI AG), following the manufacturer’s recommendations. Isolated cells were kept in PKD lysis buffer from the RNeasy FFPE kit at –80ºC until RNA was isolated.

### Immunohistochemistry

Paraffin-embedded sections (4 μm) were cut in a microtome and deparaffinated with Histolab Clear (Histolab Products, Västra Frölunda, Sweden). Antigen retrieval was performed in 10 mM citric acid pH 6.0 containing 0.05% Tween 20 (Sigma-Aldrich, St. Louis, MO) in a 2100 Retriever (Aptum Biologics, Hampshire, United Kingdom). Samples were incubated with peroxidase 1 and blocked in Background Sniper (both from Biocare Medical, Pacheco, CA). Anti-Ki67 (GTX16667; GeneTex, Irvine, CA), anti-HLA-DMA (HPA012750; Atlas Antibodies, Bromma, Sweden), anti-CD1a (M3571; Dako-Agilent, Santa Clara, CA), rabbit or mouse IgG isotype antibodies (Thermo Fisher Scientific), secondary donkey anti-rabbit IgG biotin conjugated antibody (ab6801-500; Abcam, Cambridge, United Kingdom), and secondary goat anti-mouse IgG biotin conjugated antibody (ab6788; Abcam) used to stain the samples in PBS with 0.1% bovine serum albumin (Sigma-Aldrich). The avidin/biotin-complex kit and DAB peroxidase substrate kit were used to develop the staining (Vector Laboratories, Burlingame, CA). Dehydration was carried out before mounting the slides with EcoMount (Biocare Medical). Images were acquired on an Olympus BX51 microscope.

### Reverse-Transcription qPCR

Biopsies preserved in AllProtect were homogenized in RLT buffer from the RNeasy Mini Kit supplemented with 1% 2-mercaptoethanol in a TissueLyser II instrument (all from Qiagen). Total RNA from homogenized biopsy samples was isolated using the RNeasy Mini Kit following the manufacturer’s instructions. Total RNA from frozen blood samples was isolated using TRIzol reagent (Thermo Fisher Scientific) in a 10:1 dilution (TRIzol:blood) following the recommended protocol for RNA isolation and subsequently cleaned with RNAeasy Mini Kit. RNA was quantified using a NanoDrop ND-2000 and reverse-transcribed with a High Capacity cDNA Reverse Transcription Kit (all from Thermo Fisher Scientific). Relative gene expression was quantified by RT-PCR with iTaq Universal SYBR Green Supermix (Bio-Rad, Hercules, CA) following the manufacturer’s instructions and using the primer pairs in Supplementary Table 6. Primers were designed to amplify all transcript coding variants of the selected gene in the Reference Sequence (RefSeq) collection of the National Center for Biotechnology Information (Bethesda, MD), taking the longest transcript sequence as a reference, with primers annealing in different exons for all transcript variants using Primer3Plus v2.4.2 software.^71^ Quantitative analysis was carried out in a CFX96 Touch Real-Time PCR detection system (Bio-Rad) using the relative quantification –ΔCt method. Hypoxanthine phosphoribosyltransferase (*HPRT*) *1* was used as a reference gene, and each sample was analyzed in duplicate.

### Statistical Analyses

Ki67 median percentage of staining in crypts from human colonic samples were analyzed with the nonparametric Kruskal-Wallis test when different groups were compared among each other. The nonparametric Wilcoxon test was used to compare paired samples from CC patients before and during treatment. Quantitative PCR data (–ΔCt values) were analyzed with the nonparametric Kruskal-Wallis test and Mann-Whitney test when different groups were compared and with the nonparametric Wilcoxon test when paired samples from CC patients before and during treatment were compared, and adjusted according to Benjamin-Hochberg FDR. Statistical analyses were performed and plotted in GraphPad Prism v8.0.1 (GraphPad, San Diego, CA, USA) or R.

All authors had access to the study data and reviewed and approved the final manuscript.
